# Supplementary material for: Group B streptococcal colonization: Prevalence and impact of smoking in women delivering term or near term neonates in a large tertiary care hospital in the southern United States
Source: PLoS One. 2020 Sep 17;15(9):e0239294. doi: 10.1371/journal.pone.0239294 (PMC7498066; doi:10.1371/journal.pone.0239294)
Supplement: Attachment — Submitted filename: GBS. RESPONSES TO REVIEWER of PLoS ONE.docx [file pone.0239294.s003.docx]

RESPONSES TO REVIEWER of PLoS ONE: Group B Streptococcal colonization: prevalence and impact of smoking in women delivering term or near term neonates in a large tertiary care hospital in the southern United States

RESPONSE TO SPECIFIC ACADEMIC EDITOR COMMENTS

Thank you for allowing us to rewrite our manuscript titled above following your recommendations and those of the Reviewer.

1) The PLoS ONE’s style requirements were followed
2) Consent Issues: As stated in out manuscript (page line ) the manuscript was reviewed as expedited and no consent was required since this was purely retrospective data from the HER. There was no interaction between the subjects and the researchers in any way, hence the need for consent was waived by the IRB of the Virginia Commonwealth University.
3) EHRs were assessed variously on throughout May 1 to July 31 of 2011, and subsequently same dates through 2019
4) A caption of Fig 1 and Fig 2 included
5) We have included the Tables as part of the main manuscript
6) Table 3 has been referenced in the main manuscript. Thanks for spotting that error
7) Captions for the Supporting Information files at the end of the manuscript have now been included.

RESPONSES TO REVIEWER’s COMMENTS

We have endeavored to follow the STROBE checklist while rewriting this manuscript as suggested by the Reviewer # 1. We furthermore inserted line numbers to facilitate comments and feedback

TITLE

We have changed the abbreviation “GBS” to its expanded form “Group B Streptococcal”. We thank the Reviewer for pointing out this error.

INRODUCTION

- We have reorganized the Introduction: In paragraphs 1 and 2 we have restated the importance of GBS colonization in causing GBS disease worldwide. In paragraph 3 we have stated that one of the risk factors for GBS colonization not adequately explored is tobacco smoke exposure during pregnancy. If this is found to be an independent risk factor, then this may be one additional reason why women may benefit from refraining from smoking during pregnancy
- Paragraph 1. We have added a very important reference to the opening statement “Maternal group B streptococcal (GBS) colonization is one of the most important risk factors for GBS disease in neonates” (Verani et al). We thank the Reviewer for pointing this out.
- Paragraph 2. We have deleted the first sentence in this paragraph but we have left the rest of the 2^nd^ paragraph 2 intact even though there may some information overload as stated by this Reviewer.
- As suggested by the Reviewer we have remove a controversial statement about lack of studies on sociodemographic risk factors associated with GBS colonization. We have rewritten this 3^rd^ paragraph to emphasize ONLY the lack of studies on tobacco smoking during pregnancy as a risk factor for GBS colonization. We thank the Reviewer for pointing this out.

METHODS

- Yes this was a convenience sample selected by the authors form EHRs between May and July of each year.
- Abbreviation issues corrected
- We have endeavored to the best of our ability to check the grammar and spellings as suggested by the Reviewer.
- We have added the word “statistically” in the sentence since only statistically significant variables in the univariate analysis (p<0.05) were included in the multivariate analysis (This is stated see last sentence last paragraph of Data Analysis).
- STROBE checklist: We have endeavored to rewrite and include all items of the checklist although some of the items may not necessarily be in the same order as in the STROBE checklist. We have clearly stated that only women of term or near term neonates were included (35+ week gestational age). All preterm neonates <35 weeks gestational age were excluded.

RESULTS

- We agree with the Reviewer that the Results section may be overloaded with information already in the Tables. We have therefore rewritten the Results section summarizing only the most important findings.
- The Reviewer suggested that we remove the 67 subjects with unknown GBS status in the analyses. This has been done and the sample size is now 736 subjects. The analysis has been redone but mainly affects Table 1 as can be seen. Table 2 and 3 remain unchanged since these tables only took account of the 736 subjects with known GBS status.
- The Reviewer wanted to know why we categorized maternal age (and tobacco smoke) in two different ways. As stated in our Methods section we did a trend analysis only of all the statistically significant independent predictors of GBS colonization in the univariate analysis. In the multiple logistic regression analysis, all variables were dichotomized for simplicity.
- The Reviewer felt trend analyses and Fig 1 and 2 may not be needed. However we feel that a trend analysis gives the average reader additional insight on how smoking and maternal age affect GBS colonization. The Figures clearly show that maternal age is only predictive of GBS among the non-smoking women regardless of age group.
- Footnote of Table 2 has been inserted
- The Reviewer suggests that this study should be labeled as a cohort. But because all the data were retrospectively collected from EHRs without any interactions with the subjects this is still a retrospective study and we still think Odds Ratios rather than relative risks should be calculated.

DISCUSSION

- We are very thankful for this Reviewer’s appreciation of our discussion in this study.
- We have followed the Reviewer’s suggestion to avoid any finding statements and analysis result have been deleted these (e.g P of Trend=0.02)
