# Supplementary material for: Group B streptococcal colonization: Prevalence and impact of smoking in women delivering term or near term neonates in a large tertiary care hospital in the southern United States
Source: PLoS One. 2020 Sep 17;15(9):e0239294. doi: 10.1371/journal.pone.0239294 (PMC7498066; doi:10.1371/journal.pone.0239294)
Supplement: Attachment — Submitted filename: RESPONSES TO GBS PLoS One ACADEMIC EDITOR AND REVIEWER.docx [file pone.0239294.s005.docx]

RESPONSES TO THE ACADEMIC EDITOR AND REVIEWER

We have checked the spelling mistakes and corrected lines 88 and 89: “out” is inserted between “carried” and “in” to read “carried out in” Also in a large “hospital.in Virginia” now becomes “hospital in Virginia”. Thus the period between “hospital” and “in” is deleted

Table 2. We have deleted the asterisk “*” from this Table (see “Tobacco Smoking (No of cigs/day)” and “Race”. Since we have reanalyzed the data there is no longer a need for a footnote for these variables. We thank the editor for consistently spotting this error.

Lines 152-153: We have deleted this sentence completely since it was indeed rather confusing and did not make sense. We thank the reviewer for spotting this error!

Discrepancies of proportions in the text and Tables. We again thank the reviewer for spotting these errors! After reanalyzes of the data as suggested by the previous reviewer we completely forgot to go back and to very carefully edit the numbers in the texts. All these have now been carefully corrected as listed in the lines of the new “Track Changes” as noted below:

Table 1:

- Line 150 : “2%” becomes “2.6”
- Line 151: becomes “10%” becomes “9%”

Table 2:

- Line 155: 35% becomes “32%” and “53%” becomes “50%”.
- Line 156: “63%” becomes “61%” .
- Line 157: “P of Trend= 0.003” becomes “P of Trend=0.001” .
- Line 159: “P=0.006” becomes “P=0.004”.
- Line 169: Pacific Island Americans (22%)” becomes “22.5%”.
- Lines 169-170: “White Americans (30.1%)” now becomes “(30.6)”

Data Analysis : Lines 140-141: We have deleted the portion on X^2^ analyses and left it with Fischer’s exact test since this is what was used in the analyses throughout

Table 1. “Mother Smoke during Pregnancy? “ the numbers have now been corrected and they add up to 736 not 803

Table 3. This Table has been revised as suggested by the academic reviewer: Unadjusted ORs have therefore been added to the adjusted ORs in the previous analysis and heading of the Table has been updated.

Conclusion: Because there is confusion about the use of the word “high”, the authors have decided to simply delete the use of this word. The conclusion has therefore been slightly reframed.

We very much appreciate the academic reviewer for their thorough editing of our manuscript and their suggestions for improvement. We hope you can publish it in its present form.

Please, do not hesitate to let us know if we can further improve our manuscript fit for publication in your esteemed journal.

Philip Kum-Nji, MD, MPH

(On behalf of the authors)
