# Supplementary material for: Group B streptococcal colonization: Prevalence and impact of smoking in women delivering term or near term neonates in a large tertiary care hospital in the southern United States
Source: PLoS One. 2020 Sep 17;15(9):e0239294. doi: 10.1371/journal.pone.0239294 (PMC7498066; doi:10.1371/journal.pone.0239294)
Supplement: Attachment — Submitted filename: Responses to the Academic Editor PLoS One.docx [file pone.0239294.s007.docx]

Responses to the Academic Editor PLoS One

1) We have added an Ethics Statement at the beginning of the METHODS section
2) We have, as requested, under the Ethics Statement, added a statement on how and when the EHRs were accessed during the study period. The IRB allowed the study authors full access to the EHRs of the Mother-Newborn dyad throughout the study period.

We hope you responded to your satisfaction but please, let us know if further issues need to be addressed.

Philip Kum-Nji, MD, MPH
