# Supplementary material for: Group B streptococcal colonization: Prevalence and impact of smoking in women delivering term or near term neonates in a large tertiary care hospital in the southern United States
Source: PLoS One. 2020 Sep 17;15(9):e0239294. doi: 10.1371/journal.pone.0239294 (PMC7498066; doi:10.1371/journal.pone.0239294)
Supplement: Attachment — Submitted filename: Reviewer comments.docx [file pone.0239294.s002.docx]

**PONE-D-20-15744**

**GBS colonization: prevalence and impact of smoking in women delivering term or near term neonates in a large tertiary care hospital in the southern United States**

This manuscript describes the relationship between smoking during pregnancy with group B streptococcal (GBS) colonization among women of term and near term neonates. While the manuscript is of some interest, I do not think the manuscript in its current form is of sufficient interest and quality to warrant publication in PLOS ONE.

I suggest the authors to look for STROBE check list for cross-sectional studies to ensure reporting is complete and transparent.

<https://www.strobe-statement.org/fileadmin/Strobe/uploads/checklists/STROBE_checklist_v4_cross-sectional.pdf>

My sense is that the manuscript could be strengthened by several modest changes as outlined below. Inserting line numbers may facilitate to give comments and feedbacks.

Title

- Suggest to expand GBS long form. Otherwise, the readers may confuse with Genotyping-by-sequencing or Guillian-Barre Syndrome or etc.

INTRODUCTION

- Introduction section is not well organized and not well-written.
- Introduction section needs to include - What is the problem and its supporting details? What is the added scientific value from the study and why are you conducting it? - Both globally and locally.
- Paragraph 2 – the information is overloaded and recommended a concise statement. Some detailed information can be shown by references.
- Need to add reference for the sentence “Maternal group B streptococcal (GBS) colonization is one of the most important risk factors for GBS disease in neonates.”
- The authors described “Potential sociodemographic risk factors for GBS colonization among pregnant women have not been recently explored. Perusal of the literature within the last 2 decades demonstrates a paucity of information on other potential sociodemographic risk factors associated with maternal GBS colonization during pregnancy.” in the introduction but I find myself many papers in the references as well as online. Moreover, this does not reflect the justification of the stated hypothesis.

METHODS

- The authors mentioned “the data ….. divided almost equally yearly (i.e. almost 90 patients selected each year)”. Is this selected by the authors or by the data set?
- Abbreviate first before using it (E.g. IRB, x^2^)
- Check grammar and spelling mistake throughout the manuscript
- The authors mentioned only the significant variables were put for a multiple logistic regression analysis. What do authors mean by significant? Statistically significant? What is the cut-off point, <0.05 or <0.2?
- Many items of the STROBE check list are written; however, most of them are jumbled up under different sub heading and some are missing (inclusion criteria, exclusion criteria, etc.)

RESULTS

- The authors tend to cover all information in the text and again in the table. You should only include the most important details in the text when you also have a table.
- Any reason behind for including 67 women who outcome status were unknown as study population?
- Any reason behind for categorizing maternal age in two ways?
- Trend analysis, Figure 1 and 2 may not be necessary for testing the study hypothesis.
- Foot note is missing for * in table 2
- Tables should be structured properly (check the published PLOS ONE papers).
- To me, this is a cohort study which the authors can calculate and present relative risk (strong statistic).
- All things considered, re-analysis and rewriting of results section is necessary.

DISCUSSION

- The discussion is the best part of the manuscript.
- Repeating the finding statements and analysis result (E.g. P of trend = 0.02) should be avoided here.

CONCLUSION

- The authors concluded “GBS colonization as rather high but consistent with recent national trend” which is not clear.
