# Supplementary material for: Group B streptococcal colonization: Prevalence and impact of smoking in women delivering term or near term neonates in a large tertiary care hospital in the southern United States
Source: PLoS One. 2020 Sep 17;15(9):e0239294. doi: 10.1371/journal.pone.0239294 (PMC7498066; doi:10.1371/journal.pone.0239294)
Supplement: Attachment — Submitted filename: Reviewer comments.docx [file pone.0239294.s004.docx]

**PONE-D-20-15744-R1**

**Group B Streptococcal colonization: prevalence and impact of smoking in women delivering term or near term neonates in a large tertiary care hospital in the southern United States**

My sense is that the manuscript could be strengthened by minor modifications as outlined below.

- Check the spelling mistakes (E.g. LINE 89)
- Table 2 – what do you mean *? In the responses, the authors stated that footnote was inserted but I could not find it.
- Check number in LINE 152, 153 – first 736, out for 67 , then 736 again
- Discrepancies of proportion in the text and table (check carefully)
- The above errors mean that the authors should have checked every single word in the manuscript before submission. There may be more that I couldn’t find.
- The authors stated “Using x2 analysis of comparisons of proportion with Fisher’s exact test” which is not clear. Used chi-square or Fisher’s exact?
- Table 1 - Maternal smoked during pregnancy? 🡪 803 participants?
- Table 3 should be revised (check Table 4 as example from <https://journals.plos.org/plosone/article?id=10.1371/journal.pone.0176875>)
- The authors concluded “GBS colonization as rather high but consistent with recent national data” which is not clear. Why do you say high here? Is nationally already high? High compared to what?
