# Supplementary material for: Group B streptococcal colonization: Prevalence and impact of smoking in women delivering term or near term neonates in a large tertiary care hospital in the southern United States
Source: PLoS One. 2020 Sep 17;15(9):e0239294. doi: 10.1371/journal.pone.0239294 (PMC7498066; doi:10.1371/journal.pone.0239294)
Supplement: Attachment — Submitted filename: Reviewer comments.docx [file pone.0239294.s006.docx]

**PONE-D-20-15744-R2**

**Group B Streptococcal colonization: prevalence and impact of smoking in women delivering term or near term neonates in a large tertiary care hospital in the southern United States**

Comments

- I agree about adding unadjusted OR however, Table 3 in current form is UNACCEPTABLE. The readers may get confused regarding the reference category.
- There are typo errors still remained especially in the tables. The authors stated they have corrected it but not really corrected. The authors have all the RESPONSIBILITIES for checking each and every words in the manuscript correct rather than submit the revised version fast.
